# Supplementary figures and images for: RaScALL: Rapid (Ra) screening (Sc) of RNA-seq data for prognostically significant genomic alterations in acute lymphoblastic leukaemia (ALL)
Source: PLoS Genet. 2022 Oct 17;18(10):e1010300. doi: 10.1371/journal.pgen.1010300 (PMC9612819; doi:10.1371/journal.pgen.1010300)

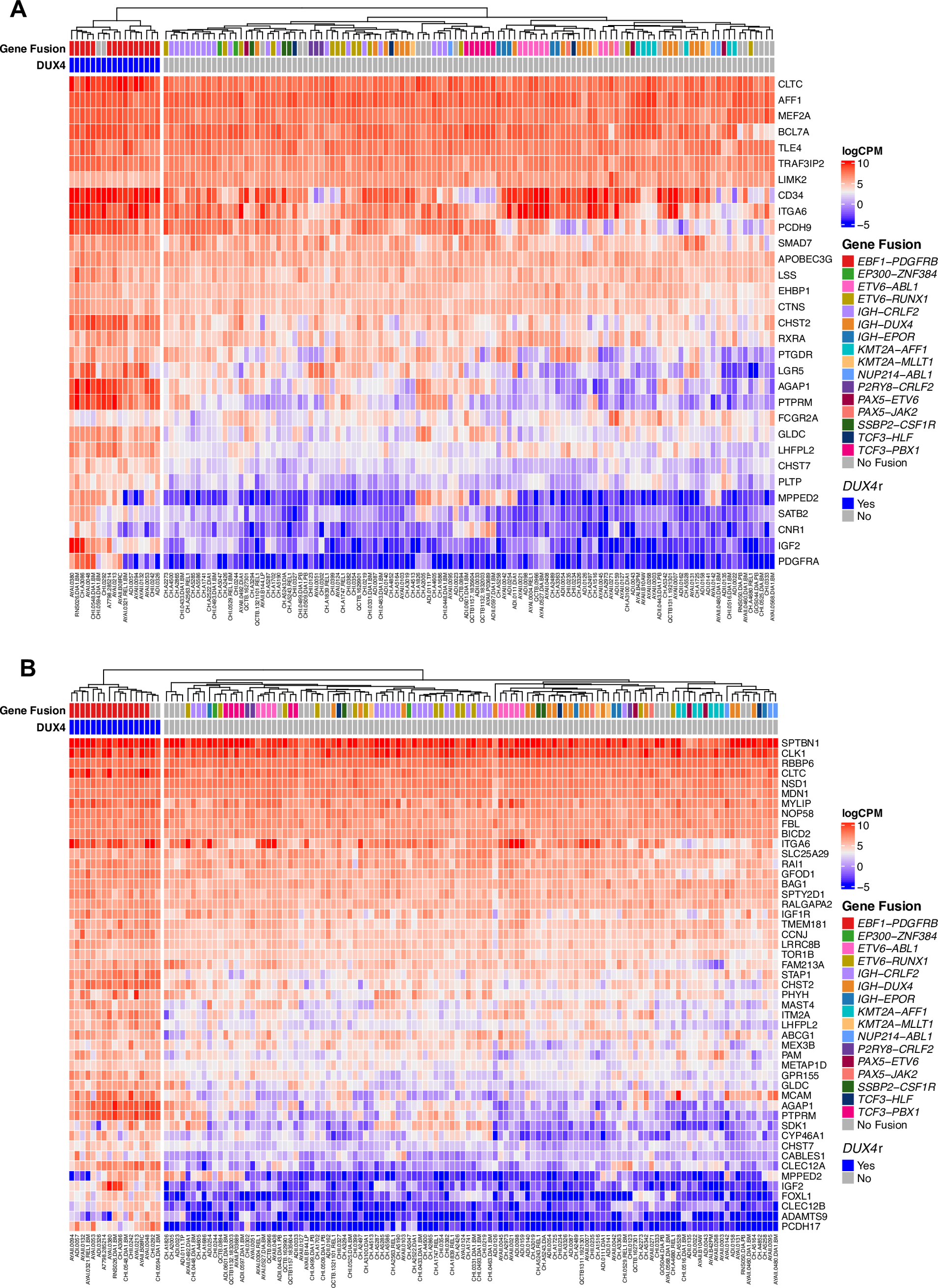

Supplement: S1 Fig — Hierarchical clustering of CPM normalised expression data using published gene sets by (A) Yeoh et al., 2002 (85) (B) Harvey et al., 2010 (86) and (C) Zhang et al., 2016 (72). Each column represents a single B-ALL patient. Driver gene fusion identified for the sample is indicated at the top of each heatmap and coloured according to the key (IGH-DUX4 fusion coloured red). Patients identified as DUX4r in the cohort are indicated with dark blue. (TIF) [file pgen.1010300.s007.tif]

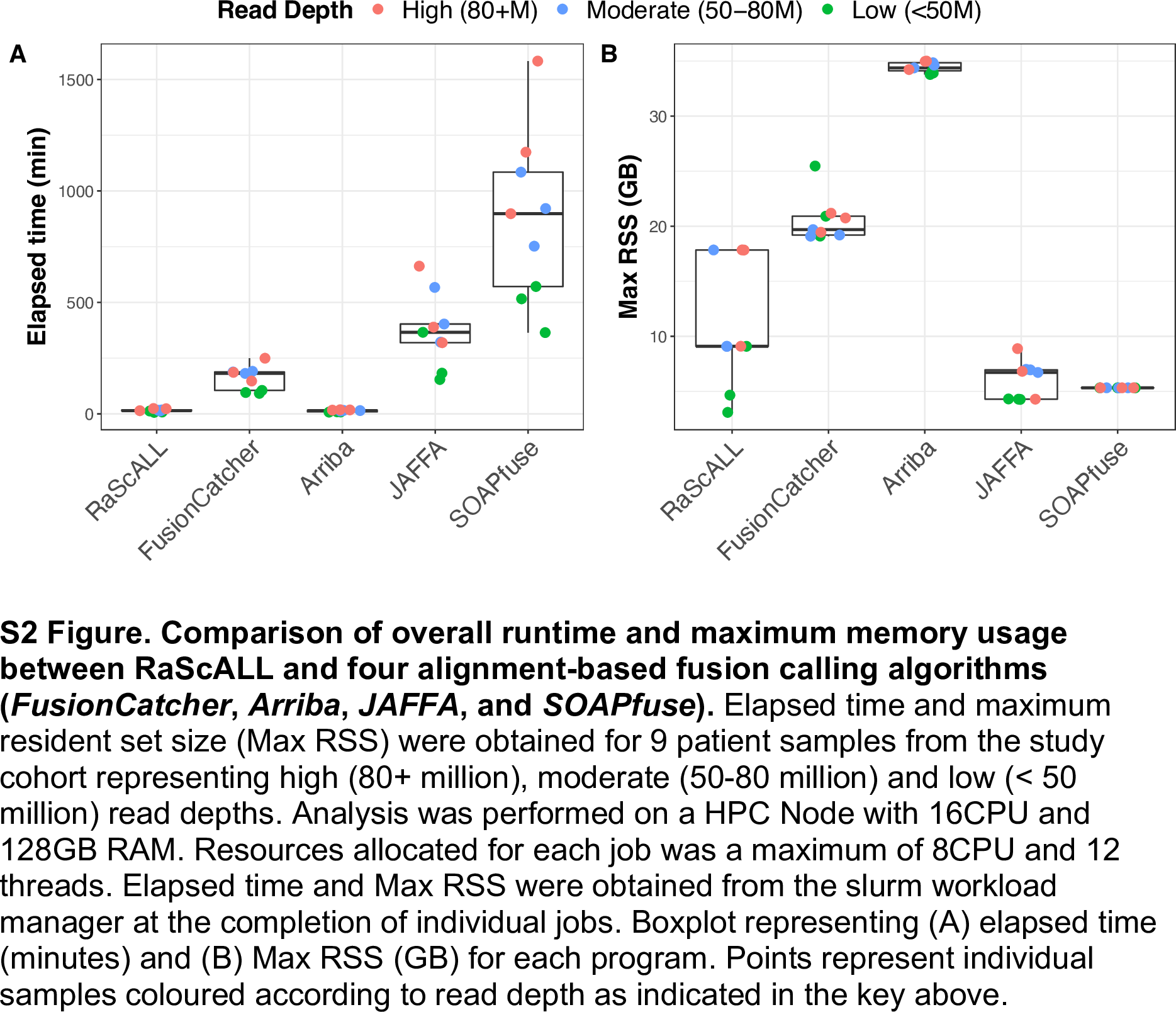

Supplement: S2 Fig — Elapsed time and maximum resident set size (Max RSS) were obtained for 9 patient samples from the study cohort representing high (80+ million), moderate (50–80 million) and low (< 50 million) read depths. Analysis was performed on a HPC Node with 16CPU and 128GB RAM. Resources allocated for each job was a maximum of 8CPU and 12 threads. Elapsed time and Max RSS were obtained from the slurm workload manager at the completion of individual jobs. Boxplot representing (A) elapsed time (minutes) and (B) Max RSS (GB) for each program. Points represent individual samples coloured according to read depth as indicated in the key above. (TIF) [file pgen.1010300.s008.tif]

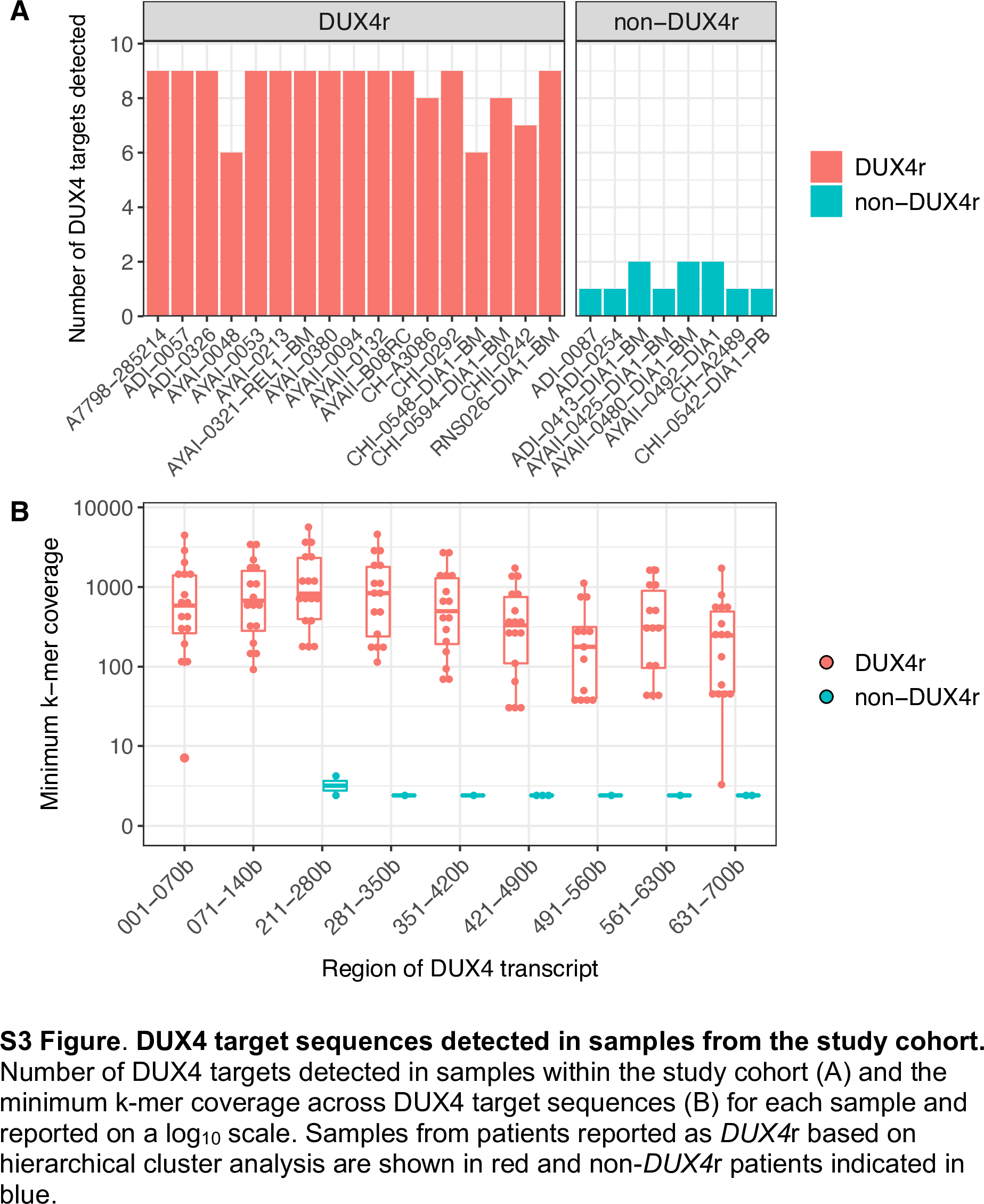

Supplement: S3 Fig — Number of DUX4 targets detected in samples within the study cohort (A) and the minimum k-mer coverage across DUX4 target sequences (B) for each sample and reported on a log10 scale. Samples from patients reported as DUX4r based on hierarchical cluster analysis are shown in red and non-DUX4r patients indicated in blue. (TIF) [file pgen.1010300.s009.tif]

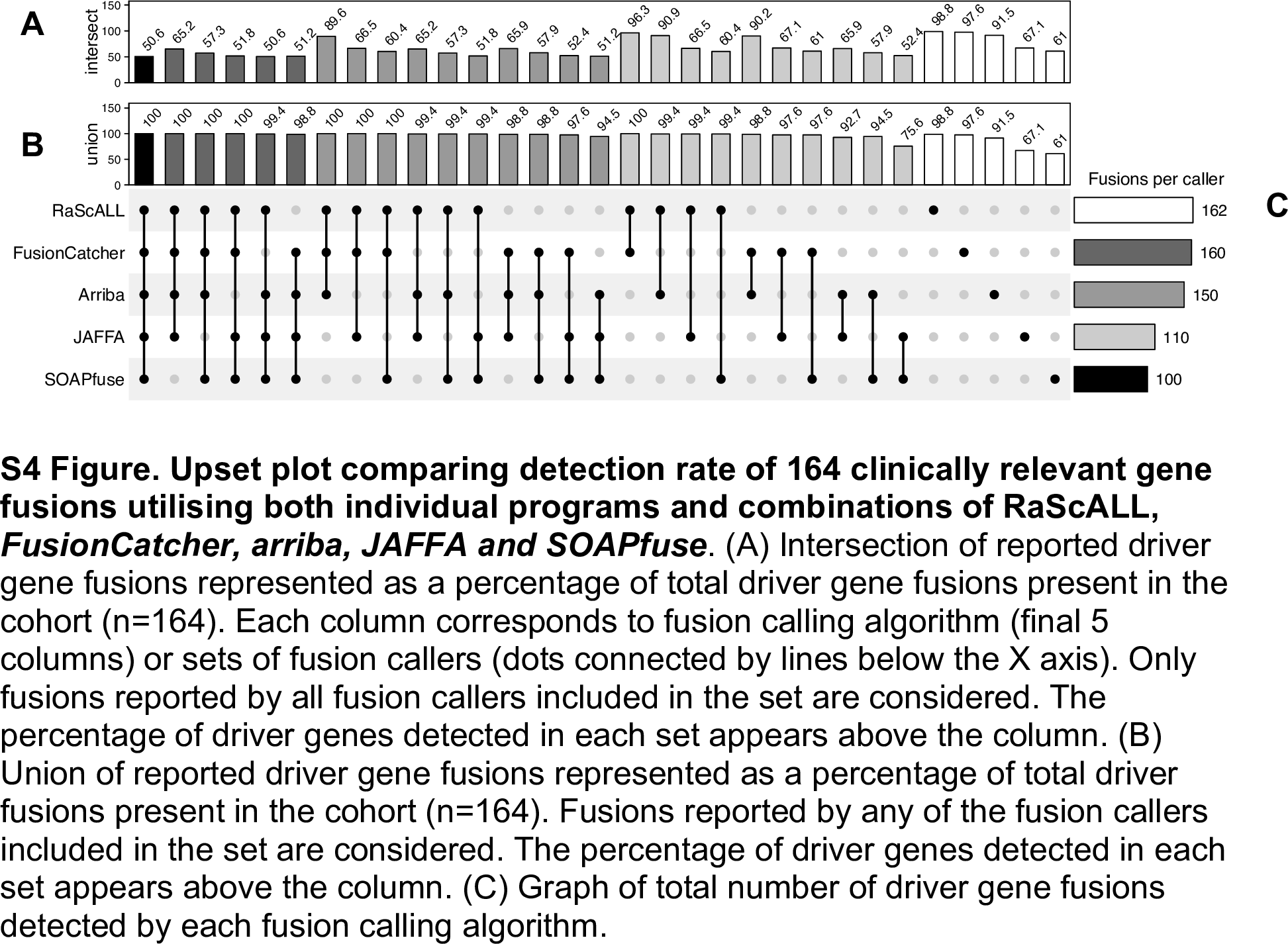

Supplement: S4 Fig — (A) Intersection of reported driver gene fusions represented as a percentage of total driver gene fusions present in the cohort (n = 164). Each column corresponds to fusion calling algorithm (final 5 columns) or sets of fusion callers (dots connected by lines below the X axis). Only fusions reported by all fusion callers included in the set are considered. The percentage of driver genes detected in each set appears above the column. (B) Union of reported driver gene fusions represented as a percentage of total driver fusions present in the cohort (n = 164). Fusions reported by any of the fusion callers included in the set are considered. The percentage of driver genes detected in each set appears above the column. (C) Graph of total number of driver gene fusions detected by each fusion calling algorithm. (TIF) [file pgen.1010300.s010.tif]
