# Supplementary material for: RaScALL: Rapid (Ra) screening (Sc) of RNA-seq data for prognostically significant genomic alterations in acute lymphoblastic leukaemia (ALL)
Source: PLoS Genet. 2022 Oct 17;18(10):e1010300. doi: 10.1371/journal.pgen.1010300 (PMC9612819; doi:10.1371/journal.pgen.1010300)
Supplement: Attachment — Submitted filename: Response_for_reviewers.docx [file pgen.1010300.s011.docx]

Dr Peter McKinnon

Cancer Genetics Editor, *PLOS Genetics*

September 18, 2022

Dear Dr. Peter McKinnon,

We present our revised submission of the manuscript “**RaScALL: Rapid (Ra) screening (Sc) of RNA-seq data for prognostically significant genomic alterations in acute lymphoblastic leukaemia (ALL)**” co-authored by Rehn *et al.* for consideration in PLOS Genetics.

Firstly, we’d like to thank the authors for the comments on the original submission of the manuscript. We have carefully considered the points made by reviewers, with the additions greatly improving the manuscript quality. In particular, we have addressed the question *“what is the real need in a clinical/diagnostic lab and whether such labs will use such a pipeline”* throughout the review comments (see below).

To this question, we acknowledge that while RNA sequencing is not utilised as a clinical diagnostic tool, it is growing in popularity due to the ability to detect cytogenetically cryptic, high-risk ALL lesions. Several recent papers have highlighted the benefits of RNA-seq in the context of clinical trials, specifically the value of sequencing data for patient subclassification. Within this context, RaScALL is a fast, lightweight sequence analysis solution for labs without extensive computational resources or analysis expertise, relying on known fusion and variant information much like a standardised molecular test. Furthermore, the ability to detect lesions from raw sequencing data means that the approach can be scaled across different computational infrastructures. including cloud solutions that are already deployed in a clinical diagnostic setting.

The manuscript has been checked thoroughly and figures validated with the PLOS Genetics PACE tool. Once again, we thank you for consideration of this manuscript.

Yours sincerely,

Dr James Breen

Chief Data Scientist, Black Ochre Data Labs

Telethon Kids Institute & Australian National University

Email: [jimmy.breen@telethonkids.org.au](mailto:jimmy.breen@telethonkids.org.au)

**Comments to the Authors:**

Reviewer #1: With this manuscript the authors describe a novel computational algorithm to identify genomic alterations that define subtypes of acute lymphoblastic leukemia from RNA sequencing. The approach uses the k-mer based targeted detection tool and known sets of ALL variants and was compared to standard alignment based pipelines. The tool appears to perform relatively well, particularly in identifying difficult to detect variants in the test cohort and a validation cohort. The approach may have some advantages in terms of required computational resources and time.

This is a potentially useful tool that appears to perform well. A disadvantage is that novel fusions may be missed as the algorithm depends on prior knowledge of variant sequences. This disadvantage is mitigated with the ability to add target sequences when they are reported. What is not discussed is how this algorithm might be implemented clinically, particularly in light of regulatory oversight of laboratory generated data that may be used for clinical decision making.

*Response:*

Thank you for highlighting this important point regarding the application of RaScALL. We agree that RNA-seq is currently not routinely used as a front-line diagnostic tool for acute lymphoblastic leukaemia in most centres. However, the increasing number of publications on this disease highlighting the cytogenetically cryptic nature of some of the most high-risk lesions indicate that RNA-seq will likely become mainstream in the future. Further, while standard diagnostic techniques remain essential for initial disease diagnosis there have been clinical trials assessing the potential for integration of RNA-seq into diagnostic and prognostic pipelines. These studies have confirmed the potential for RNA-seq to identify prognostic genomic alterations undetected by conventional methods, enabling patient subclassification and more refined risk stratification (Tran et al. 2022; Li et al. 2021). While these studies support the integration of RNA-seq as a front-line diagnostic in clinical trials, successful integration of genomic data requires the development of standardised bioinformatic tools for rapid and accurate analysis and reporting of selected genomic alterations. The research presented in this manuscript aims to address this gap.

To clarify this position, we have made the following adjustments to the text:

- Abstract (Line 60) removed the phrase ‘in a clinical setting’. Have instead stated that ‘RaScALL enables rapid identification and reporting of known risk-associated genomic alterations, allowing identification of patients who may benefit from therapeutic triage’.
- Author Summary (Line 77-78) removed the phrase ‘in a clinical setting’.
- Introduction (Line 118-119) removed the phrase ‘as a front-line diagnostic tool’.
- Results (Line 398-399 & 408) removed the phrase ‘in a clinical context’.
- Added to the discussion are references from previous studies endorsing the use of RNA-seq as a front-line diagnostic tool in clinical trials (lines 470-471).
- Incorporated into the conclusion is an explanation that RNA-seq is increasingly utilised in clinical trials for ALL and that RaScALL would be useful in this setting, where determining the presence/absence of a specific set of known genomic alterations is required for the determination of patient risk or treatment allocation. (Lines 577-581).

Reviewer #2: In this study, authors introduce a computational pipeline, RaScALL, for inferring genomic aberrations from RNA-sequencing data in ALL patients. RaScALL pipeline allows for screening and inferring gene fusions, SNVs and focal gene deletions with potential prognostic value for ALL patients. Moreover, RaScALL implements the km algorithm that conducts a pseudo-alignment rather than a direct mapping of raw RNA-seq reads to a reference genome. Authors argue that this approach is more efficient in terms of memory and computational time as it reduces the search space.

The workflow of RaScALL includes implementation of four fusion calling algorithms (FusionCatcher, SOAPfuse, JAFFA and Arriba), and the consensus of at least two of the algorithms was required for gene fusion identification (except for IGH rearrangements, IGHr).

Authors applied RaScALL in a patient cohort of 180 subjects that represent common prognostic genomic lesions in B-ALL (160), T-ALL (18) and mixed lineage leukaemia, MLL (2). They also validated RaScALL inferences via the data analysis of 100 publicly available datasets samples.
The results show that RaScALL successfully identified gene fusions in both cohorts for a fraction of computational time. Their pipeline can accommodate new reports of gene fusions and de novo variants.

I find this paper of great-quality work that makes a contribution to cancer research. Authors present a new computational tool that can benefit the clinicians for disease diagnosis in ALL patients. However, the first big question is what is the real need in a clinical/diagnostic lab and whether such labs will use such a pipeline. Let’s not forget that RNA-Seq is not used routinely for diagnostics on ALL. It is used infrequently, for example in cases that are suspected Ph-like and in most laboratories, samples are sent out for RNA-Seq to reference laboratories. Most places are probably not even sending out such samples.

Response:

We acknowledge your statement that RNA-seq is not routinely performed as a diagnostic assay and that this may limit the clinical need for our application. We refer to our response to a similar statement made by reviewer 1 (above) and the attempts made to address this concern. Namely, the removal of references in a ‘clinical setting’ (Lines 60; 77-78; 398-399 & 408) or ‘as a front-line diagnostic tool’ (Line 118-119). We have also provided an expanded explanation of the role of RNA-seq for patient subclassification supported by references in both the discussion (Lines 470-471) and conclusion (Lines 577-581).

Apart from this general comment, there are several issues that need to be addressed:

- The T-ALL cohort is small and key mutations (NOTCH1, FBXW7, PTEN etc) and not ever represented. The authors should expand the cohort and add such mutations and also ad additional events (MYC duplications, NOTCH rearrangements etc).

Response:

We agree that the T-ALL cohort is small and fewer targets were generated for the detection of T-ALL specific genomic alterations. This was highlighted in the discussion (Lines 564-567), with the explanation that there are fewer T-ALL samples available for benchmarking given that T-ALL makes up only 15-25% of ALL cases. Furthermore, T-ALL is accepted as a highly heterogeneous disease and the clinical/prognostic significance of reported alterations is less well understood (Tavakoli Shirazi et al. 2020).

The inclusion of a command-line tool for the generation of new targets allows expansion of the list of detected genomic alterations aimed to assist with this as the prognostic significance of genomic alterations becomes better defined. The major benefit to using RAscALL in this manner is the potential iterative expansion or adaptation of new targets to meet clinical reporting guidelines. *NOTCH1*, *FBXW7* and *PTEN* are common features of T-ALL and we have previously identified these in T-ALL samples (Eadie et al. 2020). Re-analysis of the T-ALL samples included in the study cohort utilising additional targets for these genes (see table 1 below) identified *PTEN* frameshift mutations in 3/3 samples and a *NOTCH1* missense mutation in 1 of 2 cases for which these had been reported by GATK Haplotype caller.

However, there remain limitations to the type of alterations that can be detected. For example, gene duplication (e.g. *MYC*) cannot be identified by RNA-seq and detection of rearrangements requires knowledge of mRNA breakpoints (we do not have this information for *NOTCH* rearrangements which appear to be quite rare).

Table 1: Additional T-ALL targets

| **Gene** | **Alteration** |
| --- | --- |
| FBXW7 | R465C/P/L |
| FBXW7 | R479Q/G |
| FBXW7 | R505C/S |
| FBXW7 | R689W |
| PTEN | T232fs/R234fs |
| PTEN | Q245fs |
| NOTCH1 | L1574P |
| NOTCH1 | L1585P/Q |
| NOTCH1 | F1592S/L1593P |
| NOTCH1 | R1598P |
| NOTCH1 | L1600P |
| NOTCH1 | L1678P/Q |
| NOTCH1 | I1680N/T/S |

- How easy is to modify RaScALL? Add mutations, rearrangements etc? How that would affect the analysis time and flow?

Response:

Adaptation of RaScALL for specific genomic alterations of interest/reporting is simply a matter of removing any targets from the directory that are not considered relevant for reporting and creating additional targets (fusion, SNV/InDel) of interest with the in-built custom_target scripts. Once a user has generated a text file containing the lesions of interest (including gene_name(s), transcript_identifier, amino_acid_position(s)/exon_breakpoint) the associated targets can be generated by RaScALL within a few minutes. RaScALL can subsequently be run against the user-specified target set without alteration to the workflow.

Complete instructions for the creation of additional targets are available on the GitHub page from which RaScALL can be accessed and installed. An expanded explanation of how the additional targets for the validation cohort were created has been added to the methods (lines 635-641). The runtime for the creation of these additional targets was less than 3 mins (added to the manuscript text lines 405-406).

- What is the cost of running RaScALL per sample?

Response:

This is a great question, although cost can be difficult to estimate given the different potential computing infrastructures that are available and run-time factors such as available memory, sample read length and sequencing depth. The mean runtime per sample from the study cohort was 16.8 minutes (read length 75bp, mean read depth 72.4M reads). This was dependent on using 12threads for jellyfish and memory requirements of ~18GB RAM, with km specifically requiring far less memory than the jellyfish steps. Based on the benchmarking results we did in this study, the average running cost (AUD$) of RaScALL on cloud computing infrastructure would be $0.31 per sample, with the highest coverage/read-length sample costing ~$0.45 per sample (24 mins). This estimate is based on a $1.12 per hour cost of a virtual machine (16vCPU/64Gb RAM/40GB persistent disk) running on google cloud infrastructure (restricted to the Australian zone for clinical samples). In comparison, the highest coverage/read-length sample running the slowest standard fusion caller (SOAPdenovo) on the same machine would be $29.53

Given that costs will additionally fluctuate depending on region and provide these exact costs were not added to the manuscript. However, we have included a general statement in both the results section (lines 255-259) and discussion (lines 542-545) explaining that the higher speed and reduced memory translate to lower computational costs for sample analysis.

- Lines 132-134: Add the citation of the km algorithm.

Response:

The citation for km has been included on line 133 and is repeated on line 138.

- Lines 156-161: There are some inconsistencies here when describing the study cohort. E.g., You have merged the ALL, MLL patients for the data analysis as I can see in the Methods section and Figure 1. But this is not clear here. You should also explain here that there was a validation cohort, different ALL subtypes etc.

Response:

We apologise for the confusion in our description of the study cohort. All patient samples received and considered for inclusion in this analysis are acute leukaemia. The two samples initially listed as MLL have been subsequently identified as acute bi-phenotypic leukaemia and acute undifferentiated leukaemia. We have clarified this in the text (lines 159-160) and separated these samples in Figure 1. A statement explaining that a separate B-ALL validation cohort was additionally employed to validate results has also been included in this section (lines 157-158) with additional information regarding the validation cohort supplied in the results when describing the effectiveness of RaScALL to detect lesions in the validation cohort (lines 407-410).

- Figure1: It would be interesting to see how the identification of genetic fusions change in ALL patients in different disease stages (patients at Diagnosis vs Relapse disease stages). Are there any differences? Maybe consider to add the time point here? Also, you should include here the results from the study and validation cohorts together.

Response:

The same gene fusion detected at diagnosis is present at relapse with no difference in how these alterations are detected. We have added to Figure 1 the timepoint at which the sample analysed was taken.

The validation cohort consists of samples from a previously published study. The detected gene fusions and ALL associated single nucleotide variants have already been presented as part of this earlier publication (Gu Z, Churchman ML, Roberts KG, Moore I, Zhou X, Nakitandwe J, et al. PAX5-driven subtypes of B-progenitor acute lymphoblastic leukemia. *Nat Genet*. 2019 Feb 14;51(2):296–307.)

- Line 169: You need to explain why IGHr were excluded. It seems that this is explained later as “regions with highly variable breakpoint locations” (Line 197). But you should elaborate more on this, and add a citation.

Response:

We again apologise for the confusion. *IGH* rearrangements (*IGH*r) were not excluded but are more difficult to detect by standard alignment-based fusion calling algorithms. We, therefore, do not hold to the same criteria when calling *IGH*r as we do for detection of other gene fusions (i.e. do not require a consensus between two or more callers). Instead, any *IGH*r that is detected by at least one fusion calling algorithm is verified by other means (FISH, gene expression profiling, manual inspection etc). We have edited the text on lines 173–178 to clarify this for the reader and included citations regarding the under-reporting of *IGH* rearrangements.

- Lines 171-172 and Lines 397-398: You mention that your study cohort contained patient of high sequencing coverage (mean 70M PE reads). How is the performance of RaScALL affected by the read coverage? Provide minimum read coverage or suggestions for the user to deal with such patient cases.

Response:

This is an important point as genomic alterations which are transcribed at lower levels will be detected less reliably by RaScALL (although this is true of many algorithms). For SNVs/InDels a minimum of 5 reads spanning the target region must contain the altered allele to be detected by RaScALL. For an SNV with a variant allele frequency of 0.2, a minimum depth of 25X across this region would therefore be required. This information has been added to the discussion (lines 524-526) when describing the limitations of RaScALL for the detection of cooperating mutations, along with a suggestion that the user performs follow-up analysis with additional tools (e.g., GATK) in cases where this requirement is not met.

- Line 189: are the identified pathogenic SNVs first-time reported here (thanks to RaScALL) or are these based on known SNVs?

Response:

Pathogenic SNVs described in the manuscript are all known/previously reported. This point has been clarified in the figure legend (lines 198-199).

- Lines 250-251: This information is partially stated as part of the beginning in the Results section (Lines 156-160); consider merging these two parts.

Response:

Thank you for highlighting the repetition in the text. We have edited the paragraph to reduce overlap but still orient the reader to the analysis being described in this section (lines 269-272).

- Line 304: To add citation about MLPA analysis.

Response:

Citations regarding the use of SNParray or MLPA for detection of *IKZF1* deletions have been added to the text (Lines 325-327).

- Line 326: Caption should be more self-explanatory. E.g., explain a little bit what MLPA analysis is. Are the results here related to the detection of IKZF1 intragenic deletions by using RaScALL and comparing them to the MLPA analysis?

Response:

Thank you for identifying the lack of clarity in the figure legend. We have expanded the caption to provide a more detailed explanation of how the analysis was performed and what the plots represent (lines 349-363).

- Line 385, Figure 4: You should show the overall performance, accuracy and run time of RaScALL pipeline comparing to the other methods (FusionCatcher, SOAPfuse, JAFFA and Arriba), and how these change as you get concordance from 2, 3 or all 4 methods.

Response:

To clarify, the validation dataset represented in Figure 4 was not processed through the same fusion calling pipeline (using FusionCatcher, SOAPfuse, JAFFA and Arriba) as samples from the study cohort. Instead, samples in the validation cohort were analysed by RaScALL and the genomic alterations reported by RaScALL were then compared to the genomic alterations reported in the initial study by Gu et al., 2019. We apologise for this confusion and have edited the text on lines 412-416 to make this more apparent.

A comparison of runtime and resource allocations between RaScALL and other *de novo* fusion calling algorithms was omitted from the initial manuscript as it was not considered a fair comparison. RaScALL can only detect a limited set of alterations (those with available targets) and cannot detect novel events, unlike the other fusion calling algorithms mentioned. However, to highlight the advantages of targeted analysis where a finite list of genomic alterations is to be assessed we have added to the supplementary data a benchmarking assessment of runtime and memory requirements for RaScALL and the other 4 fusion calling algorithms (S5 Fig). Important points from this benchmarking assessment have been added to the results section describing memory and run-time evaluation (lines 252-255) and the discussion (lines 532-537). Details for completing this benchmarking analysis have also been added to the methods (lines 715-724).

We have additionally included, as a supplementary figure, an upset plot comparing the detection frequency of driver gene fusions in the study cohort when seeking concordance between 2, 3, 4 or all 5 methods (S8 Figure, referenced line 318).

- Lines 430-431: Maybe, it would be useful for researchers/clinicians to get a prognostic risk score or a label that would indicate ALL subtype and/or disease stage based on the identification of the genetic lesions, that would help patient stratification and indicate progression of the disease.

Response:

We appreciate that a prognostic risk label would be useful in some cases, but this ignores the complex nature of risk-assessment in ALL. RaScALL was designed as a bioinformatic tool to assist with the detection of genomic alterations of potential clinical significance, not to represent a comprehensive solution to the very complex problem. Overall prognostication would require parameters beyond mere genomic information (e.g. patient age, white blood cell count (WCC), cytogenetics and initial response to treatment). It would therefore not be appropriate for us to suggest information from RaScALL alone to be adequate to predict outcomes.

Eadie, Laura N., Jacqueline Rehn, Susan L. Heatley, Barbara J. McClure, Caitlin Schutz Schutz, James Breen, Timothy P. Hughes, David T. Yeung, Rosemary Sutton, and Deborah L. White. 2020. “Next Generation Genomic Analyses in T-ALL Patients Identify Recurrent and Novel Genomic Abnormalities.” *Blood* 136 (Supplement 1): 13–14.

Li, Zhenhua, Shawn Hsien Ren Lee, Winnie Hui Ni Chin, Yi Lu, Nan Jiang, Evelyn Huizi Lim, Elaine Coustan-Smith, et al. 2021. “Distinct Clinical Characteristics of DUX4- and PAX5-Altered Childhood B-Lymphoblastic Leukemia.” *Blood Advances* 5 (23): 5226–38.

Tavakoli Shirazi, Paniz, Laura N. Eadie, Susan L. Heatley, Timothy P. Hughes, David T. Yeung, and Deborah L. White. 2020. “The Effect of Co-Occurring Lesions on Leukaemogenesis and Drug Response in T-ALL and ETP-ALL.” *British Journal of Cancer* 122 (4): 455–64.

Tran, Thai Hoa, Sylvie Langlois, Caroline Meloche, Maxime Caron, Pascal Saint-Onge, Alexandre Rouette, Alain R. Bataille, et al. 2022. “Whole-Transcriptome Analysis in Acute Lymphoblastic Leukemia: A Report from the DFCI ALL Consortium Protocol 16-001.” *Blood Advances* 6 (4): 1329–41.
